# Cucurbitacin D Reprograms Glucose Metabolic Network in Prostate Cancer

**DOI:** 10.3390/cancers11030364

**Published:** 2019-03-14

**Authors:** Mohammed Sikander, Shabnam Malik, Neeraj Chauhan, Parvez Khan, Sonam Kumari, Vivek Kumar Kashyap, Sheema Khan, Aditya Ganju, Fathi T. Halaweish, Murali M. Yallapu, Meena Jaggi, Subhash C. Chauhan

**Affiliations:** 1 Department of Pharmaceutical Sciences, University of Tennessee Health Science Centre, Memphis, TN 38163, USA; msikande@uthsc.edu (M.S.); smalik@uthsc.edu (S.M.); nchauhan@uthsc.edu (N.C.); skumari@uthsc.edu (S.K.); vkashya1@uthsc.edu (V.K.K.); skhan14@uthsc.edu (S.K.); adityaganju11@gmail.com (A.G.); myallapu@uthsc.edu (M.M.Y.); 2 Centre for Interdisciplinary Research in Basic Sciences, Jamia Millia Islamia, New Delhi 110025, India; parvezynr@gmail.com; 3 Department South Dakota State University, Brookings, SD 57007, USA; fathi.halaweish@sdstate.edu

**Keywords:** cucurbitacin D, PrCa, miRNAs and glucose metabolism

## Abstract

Prostate cancer (PrCa) metastasis is the major cause of mortality and morbidity among men. Metastatic PrCa cells are typically adopted for aberrant glucose metabolism. Thus, chemophores that reprogram altered glucose metabolic machinery in cancer cells can be useful agent for the repression of PrCa metastasis. Herein, we report that cucurbitacin D (Cuc D) effectively inhibits glucose uptake and lactate production in metastatic PrCa cells via modulating glucose metabolism. This metabolic shift by Cuc D was correlated with decreased expression of GLUT1 by its direct binding as suggested by its proficient molecular docking (binding energy −8.5 kcal/mol). Cuc D treatment also altered the expression of key oncogenic proteins and miR-132 that are known to be involved in glucose metabolism. Cuc D (0.1 to 1 µM) treatment inhibited tumorigenic and metastatic potential of human PrCa cells via inducing apoptosis and cell cycle arrest in G2/M phase. Cuc D treatment also showed inhibition of tumor growth in PrCa xenograft mouse model with concomitant decrease in the expression of GLUT1, PCNA and restoration of miR-132. These results suggest that Cuc D is a novel modulator of glucose metabolism and could be a promising therapeutic modality for the attenuation of PrCa metastasis.

## 1. Introduction

Prostate cancer (PrCa) is one of the most common malignancies and second leading cause of cancer related deaths among American men [1]. Emergence of castration resistant PrCa (CRPC) and chemo-resistance are some of the major hurdles in managing PrCa [2,3]. Accumulating evidence has shown that cancer cells are intricately sensitive to metabolic alterations that modify the metabolic homeostasis [4,5]. To maintain a fast growing need for intermediates, cancer cells reprogram their metabolic pathways. Interestingly, it has been reported that early and advanced stages of PrCa have quite different glucose metabolism [6], and this glycolytic metabolism displays a divergent profile in androgen-sensitive and insensitive PrCa cells [7]. Furthermore, a higher glucose uptake is required for rapid proliferation of androgen-insensitive PrCa cells [8]. Glucose transporters (GLUTs) are responsible for glucose uptake in cells by a mechanism of facilitated diffusion. Recent studies have indicated that reprograming of cancer metabolism is a novel therapeutic strategy for PrCa management. These findings suggest that GLUT1 is a very important molecular target for PrCa therapy. Thus, non-toxic agents/pharmacological inhibitors which have potential to modulate glucose metabolism can be useful for PrCa management.

Natural agents have always been appreciated for the treatment of various disease including cancer because of their low or minimal toxicity. Various natural agents have shown their potential therapeutic and preventive effects in in vitro and pre-clinical mouse model systems. Recently, some natural agents have shown their anti-cancer effects via altering glucose metabolism or inhibiting the expression of glucose transporters [9]. Inhibition of glucose uptake reduces cell growth and induces apoptosis in tumor cells [10]. It has been shown that natural compounds that target different tyrosine kinases or ATP binding sites are able to inhibit glucose transporter activity [11]. It has been reported that natural compounds, including flavonoids, are able to modify glucose uptake by regulating GLUT1 expression and/or altering glucose binding to them [12].

Cucurbitacins are identified as tetracyclic triterpenoids and belongs to the Cucurbitaceae family. They are known to have diverse pharmacological activities including anti-inflammatory, antitumor and antimicrobial activities [13,14]. Among several derivatives, cucurbitacin B, D, E and I have been widely studied for their strong anticancer activities [14]. Accumulating studies have shown that they are primarily JAK-STAT inhibitors and have shown potent anti-cancer activities [15,16,17].

In this study, we have evaluated the therapeutic efficacy and underlying molecular mechanisms of Cuc D against PrCa using in vitro and in vivo model systems. The observed effect of Cuc D might be due its effect on altering glucose metabolism, suppression of PI3K/AKT signaling pathways and restoration of miR-132 expression in PrCa cells. 

## 2. Results

### 2.1. Cuc D Inhibits Growth and Induces Apoptosis Mediated Cell Death of PrCa Cells

**Figure 1:**
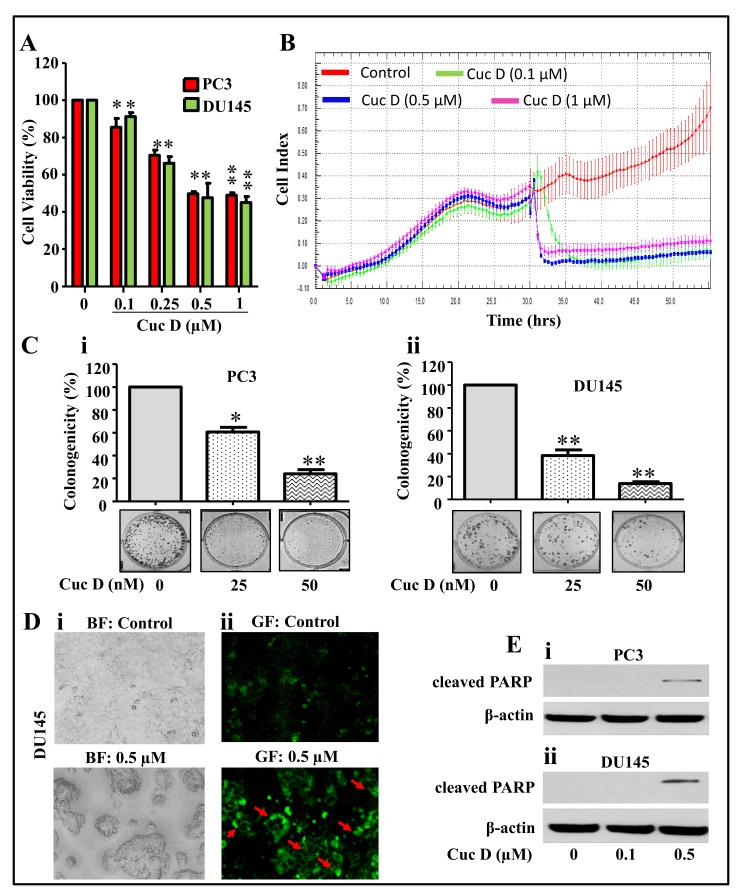
Effect of Cuc D on cell proliferation, clonogenic potential and apoptosis induction in PrCa cells. (**A**) Effect of Cuc D on cell viability of PC3 and DU145. Briefly, cells were seeded in 96 well plate and after overnight incubation, treated with indicated concentrations of Cuc D for 48 h. Cell viability was assessed by MTT assay. The bar graph represents the percent viable cells compared to vehicle treated cells. Each concentration value is the mean ± SE of triplicate well of each group. Asterisk indicate statistical significance determined by Student’s *t*-test (* *p* < 0.05 and ** *p* < 0.01). (**B**) Effect of Cuc D on cell proliferation with respect to time was also confirmed by xCelligence assay. (**C**) Effect of Cuc D on colony formation of PrCa cells. In brief, 500 cells were seeded in each well of 6 well plates. After 3 days, cells were treated with indicated concertation of Cuc D for 7 days and then media was replaced with complete growth media and colonies were obtained which were further stained with hematoxylin. Photographs were taken by UVP-gel documentation system for PC3 (**Ci**) and DU145 (**Cii**). Bar graph represents number of colonies formed in each group of PC3 and DU145 cells. Experiments were repeated in triplicate with similar results. Asterisk indicate statistical significance determined by Student’s *t*-test (* *p* < 0.05 and ** *p* < 0.01). (**D**) Effect of Cuc D on apoptosis induction of DU145 cells as determined by Annexin V staining. In Brief, 0.5 × 10^6^ cells were seeded in each well of 6 well culture plate. After 24 h, cells were treated with indicated concentrations of Cuc D and apoptosis induction was measured by Annexin V staining under fluorescent microscope. Representative images of control and Cuc D treated cells under bright field (BF) (**Di**) and green fluorescent (GF) (**Dii**). GF images (20×) represent the Annexin V stained cells as indicated by arrows. (**E**) Effect of Cuc D on protein levels of early apoptotic biomarker (cleaved PARP) in PC3 (**i**) and DU145 (**ii**) cells as determined by western blot analysis. β-actin was used as internal loading control.

In our study, we used androgen-independent PrCa cell lines, PC3 and DU145. To determine the cytotoxic potential of Cuc D in PrCa cells, an MTT assay was employed. As shown in Figure 1A, Cuc D treatment dose dependently inhibited the cell viability of PrCa cells. Next, we determined the effect of Cuc D on PrCa cell proliferation by xCELLigence and colony formation assays. Results demonstrated that Cuc D treatment (0.1–1.0 µM) inhibited proliferation of DU145 cells as reflected by a decrease in baseline cell index (Figure 1B). Cuc D treatment significantly decreased the number of colonies in both PC3 (Figure 1Ci) and DU145 cells (Figure 1Cii). The effect of Cuc D on colony formation at 25 nM concentration was more significant (*p* < 0.001) in DU145 cells compared to PC3 cells. Since we observed that Cuc D exert potent cytotoxic and growth inhibitory effects, we further examined the effect of Cuc D on apoptosis induction. PrCa cells were treated with Cuc D (0.5 µM) for 24 h and the apoptosis inducing effect of Cuc D was analyzed by Annexin V staining and Western blot analysis for cleavage in PARP protein. Our results revealed that Cuc D treatment induced apoptosis in DU145 cells as observed by enhanced Annexin V staining (Figure 1D). Western blot analysis showed that Cuc D treatment dose dependently enhanced the protein levels of cleaved PARP in PC3 (Figure 1Ei) and DU145 (Figure 1Eii) cells. These results suggest that Cuc D exhibited potent growth inhibitory and apoptosis inducing abilities in PrCa cells.

### 2.2. Cuc D Arrests Cell Cycle of PrCa Cells in G2/M Phase

**Figure 2:**
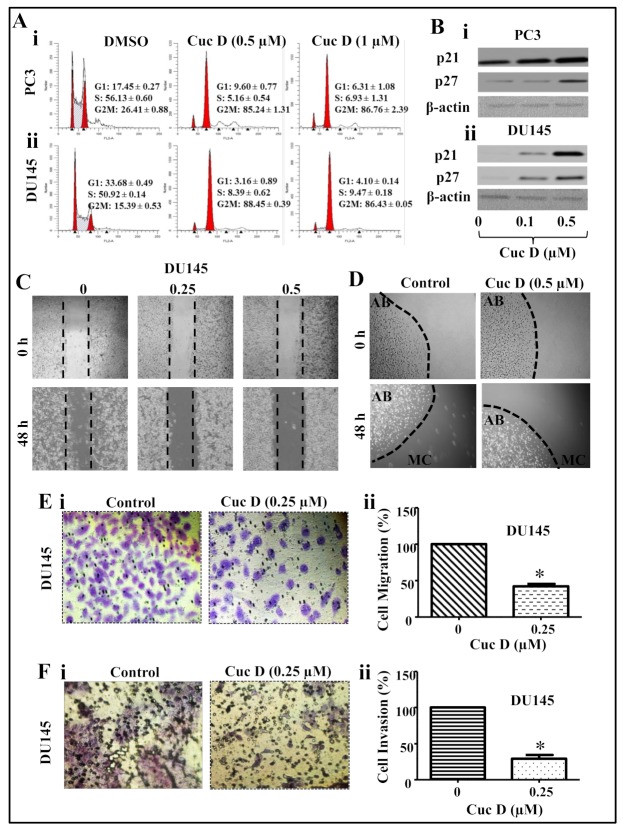
Effect of Cuc D on cell cycle progression, migration and invasive abilities of PrCa cells. (**A**) Effect of Cuc D on cell cycle distribution in PrCa cells. Cuc D arrests PC3 (**Ai**) and DU145 (**Aii**) cell cycle in G2/M phase as determined by flow cytometry. (**B**) Effect of Cuc D on protein levels of cell cycle regulatory proteins (p21 and p27) in PC3 (**Bi**) and DU145 (**Bii**) cells as determined by Western blot analysis. (**C**–**E**) Effect of Cuc D on migration of PrCa cells as determined by scratch wound, bead assay and Boyden chamber assays. (**C**) Representative images of migratory DU145 cells in control and treated groups at 0, 48 h as determined by scratch wound assay. (**D**) Illustrative images of migratory cells (MC) in control and Cuc D treated groups at 0 and 48 h as determined by agarose bead assay. (**E**) Effect of Cuc D on migration of DU145 cells as examined by Boyden chamber assay as explained in the Materials and Methods section. In brief, 18 h post-treatment of indicated concentration of Cuc D, migrated cells were fixed and counted in control and Cuc D treated DU145 cells (**Ei**). Bar graph represents the quantification of migrated DU145 cells (**Eii**). (**F**) Effect of Cuc D treatment at 18 h on invasion of PrCa cells as described under materials and methods. Illustrative images (20×) of invaded control and Cuc D treated DU145 cells (**Fi**). Bar graph represents the quantification of DU145 (**Fii**) cells. Single asterisk (*) denotes the significant value *p* < 0.05.

Cell cycle arrest is an attractive target for the management of various types of cancers [18]. Thus, to examine the effect on cell cycle distribution, PrCa cells were treated with Cuc D (0.5 and 1 µM) and analyzed by flow cytometry. Result showed a dose-dependent increase of Cuc D treated PC3 (Figure 2Ai) and DU145 (Figure 2Aii) cells in the G2/M phase. Further, to gain insight into cell cycle arrest by Cuc D, we also studied the effect of Cuc D on cell cycle inhibitory proteins (p21 and p27). As shown in Figure 2, Cuc D treatment (0.1 and 0.5 µM) dose-dependently up-regulated the expression of p21 and p27 in PC3 (Figure 2Bi) and DU145 (Figure 2Bii) cells as revealed by western blot analysis.

### 2.3. Cuc D Inhibits The Migratory and Invasive Potential of PrCa Cells

PrCa metastasis is one of the major problems for the treatment of PrCa. Thus, to examine whether Cuc D treatment inhibits the migratory potential of PrCa cells, we executed the scratch wound, agarose beads and Boyden chamber assays. Cuc D treatment showed dose dependent inhibition of migration abilities in DU145 cells (Figure 2C) in dose dependent manner. At 48 h, we observed that the wound was comparatively more filled in vehicle group than Cuc D treated group. Consistently, agarose beads assay showed that Cuc D inhibited the motility of PrCa cells (Figure 2D). Furthermore, Boyden chamber assays performed under chemotactic drive also revealed a decrease in migration of PrCa following the Cuc D treatment (Figure 2Ei,ii). Since Cuc D suppressed the motility of PrCa cells, we were further interested to investigate whether Cuc D treatment modulates invasion abilities of PrCa cells. Interestingly, results illustrated that Cuc D (0.5 µM) effectively inhibited invasion of PrCa cells (Figure 2Fi,ii). Taken together, these data suggest that Cuc D suppresses migration and invasive ability of PrCa cells.

### 2.4. Cuc D Treatment Decreases Glucose Metabolism in PrCa Cells

**Figure 3:**
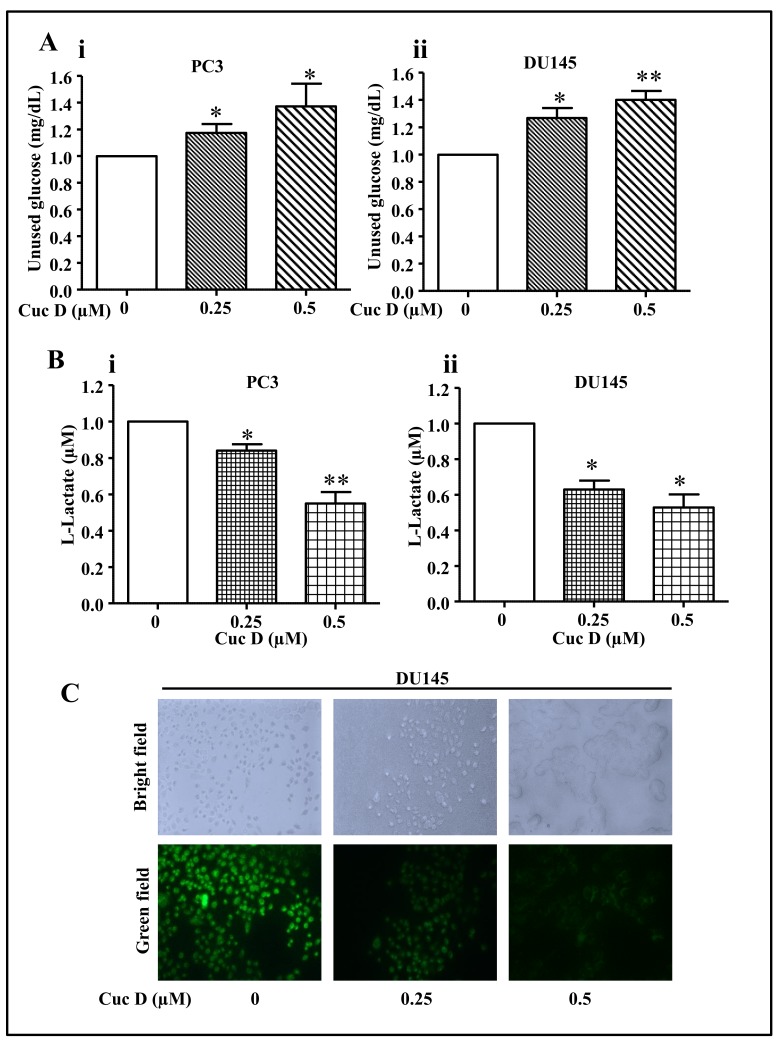
Effect of Cuc D on glucose uptake and lactate secretion by PrCa cells. (**A**) Effect of Cuc D on glucose uptake by PrCa cells as determined by glucose colorimetric assay kit (Cayman Chemicals, Ann Arbor, MI, USA). Bar graphs represent the unused glucose in PC3 (**Ai**) and DU145 (**Aii**) cells 24 h post-treatment of indicated concentrations of Cuc D. Asterisk indicate statistical significance determined by Student’s *t*-test (* *p* < 0.05 and ** *p* < 0.01). (**B**) Effect of Cuc D on lactate secretion by PrCa cells as determined by glycolysis cell-based assay kit (Cayman Chemicals, Ann Arbor, MI, USA). Bar graphs represent lactate secretion by PC3 (**Bi**) and DU145 (**Bii**) cells 24 h post-treatment of Cuc D. Asterisk indicate statistical significance determined by Student’s *t*-test (* *p* < 0.05 and ** *p* < 0.01). (**C**) Effect of Cuc D on glucose uptake as studied by fluorescence microscopy. Photomicrograph represents the uptake of fluorescent probe, 2-NBDG, by DU145 cells (20×). Briefly, cells were treated with Cuc D at indicated concentrations and uptake of 2-NBDG was analyzed by fluorescence microscopy. Green field images represent the fluorescent probe, 2-NBDG.

Metastatic prostate cancer cells have been shown to have higher glucose metabolism [19]. Since it was observed that Cuc D inhibits metastatic phenotypes of PrCa cells, we were interested to investigate whether Cuc D alters the glucose metabolic shift. Thus, effect of Cuc D on glucose uptake and lactate secretion was determined by using commercially available kits. It was observed that after Cuc D treatment, glucose uptake from media was dose dependently inhibited (Figure 3Ai,ii) while a decrease in lactate secretion (Figure 3Bi,ii) was observed as compared to vehicle group in both PC3 and DU145 cells. Furthermore, the effect of Cuc D on glucose uptake in DU145 cells was further examined by using 2-NBDG, fluorescent probe. As shown in Figure 3C, the intensity of fluorescent signal was dose-dependently decreased in Cuc D treated group as compared to vehicle treated cells. These findings suggest that Cuc D reprograms the metabolic network in PrCa cells. 

### 2.5. Cuc D Treatment Targets The GLUT1 Protein via Modulating miR-132 Expression 

**Figure 4:**
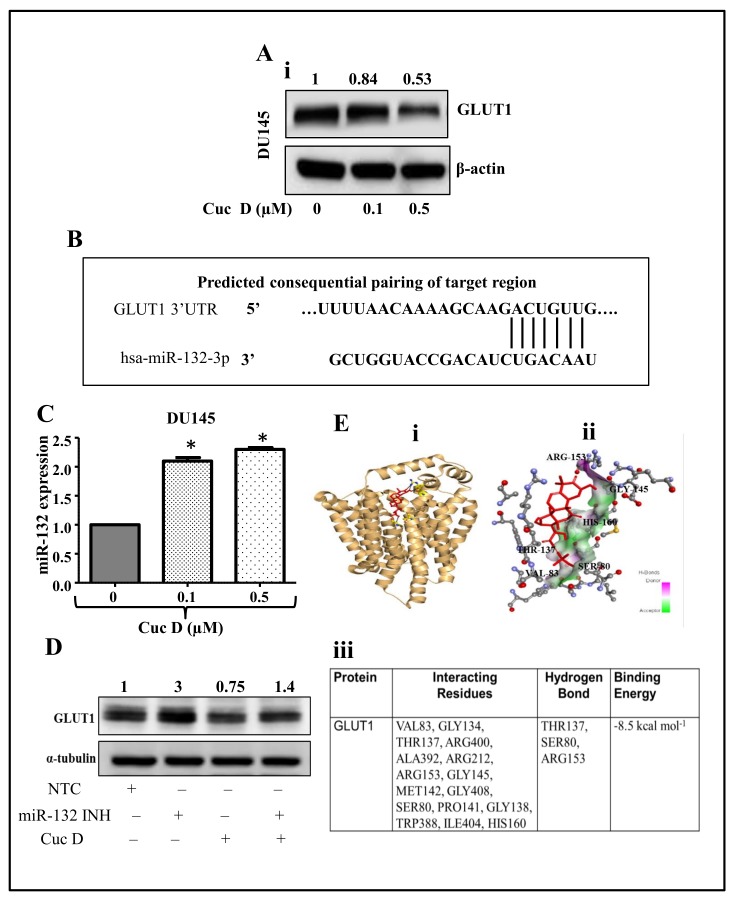
Molecular mechanism of Cuc D targeting GLUT1 in PrCa cells. (**A**) Effect of Cuc D on GLUT1 expression in DU145 cells (**A**) as examined by western blot analysis. Briefly, cells were treated with denoted concentrations of Cuc D for 24 h and total cell lysates were prepared and subjected for western blot analysis for GLUT1 protein level. β-actin was used as an internal loading control. Values shown above the blot are densitometric analysis of GLUT1 blot normalized with β-actin using GelQuant software. (**B**) Putative miR-132 binding sites in the SLC2A1 3′UTR region of GLUT1. Seven bases (192 through 198) of the SLC2A1 3′UTR are perfect matches (seed sequences) for miR-132 binding. (**C**) Effect of Cuc D on the expression of miR-132 in PrCa as determined by qPCR analysis. RNU6B was used as an internal control. Asterisk (*) denotes the significant value *p* < 0.05 when applied student’s *t*-test. (**D**) Effect of Cuc D on GLUT 1 expression after transfection of the cells with miR-132 inhibitor as determined by western blot analysis. α-tubulin was used an internal loading control. (**E**) Molecular docking studies of Cuc D with GLUT1 as determined by AutoDock 4 package. Cartoon view of Cuc D docked with GLUT1 protein (**Ei**). Stereo view of GLUT1 binding with Cuc D, showing hydrogen bond donors and acceptors residues around components (**Eii**). Table depicting the GLUT1 residues interacting with Cuc D (**Eiii**).

We next studied the effect of Cuc D treatment on protein expression of GLUT1 by Western blot analysis. It was observed that Cuc D treatment suppresses the GLUT1 expression in DU145 (Figure 4Ai,ii) cells in dose-dependent manner. It is reported that expression of GLUT1 is directly regulated by miR-132 (Figure 4B) [20]. Therefore, to further understand the underlying mechanisms, we next evaluated the expression of miR-132 following the Cuc D treatment. It was observed that Cuc D treatment showed a significant (*p* < 0.05) increase in the expression of miR-132 in PrCa cells (Figure 4C) as determined by qPCR analysis. Additionally, silencing the expression of miR-132 by using inhibitor concomitantly restored the expression of GLUT1 in Cuc D treated DU145 cells (Figure 4D). These results suggest that Cuc D treatment inhibits the expression of GLUT1 via restoration of miR-132 expression in PrCa cells.

### 2.6. In Silico Studies Displays Cuc D Proficiently Bind with GLUT1

In order to study the binding pattern of Cuc D with GLUT1, a docking analysis was performed. Results showed that ligand Cuc D proficiently binds with GLUT1 and binding free energy for this complex was −8.5 kcal/mol. Figure 4Ei,ii shows that Cuc D spatially fit into the binding pocket of GLUT1. Analysis showed that Cuc D interacts with several residues and is involved in hydrogen bonding with THR137, SER80 and ARG153 (Figure 4Eii). These results suggest that Cuc D interacts with GLUT1 with both electrostatic (H-bonding) as well as hydrophobic interactions.

### 2.7. Cuc D Inhibits The Expression of Key Signaling Components Involved in Glucose Metabolism and Cell Survival in PrCa

**Figure 5:**
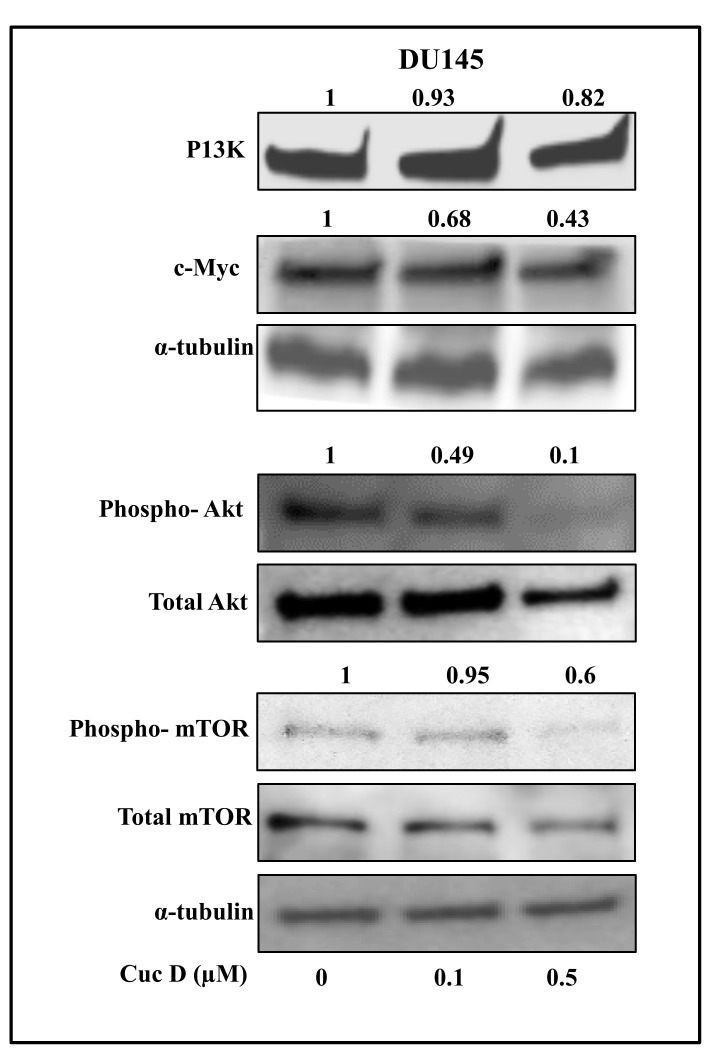
Effect of Cuc D on key effectors involved in glucose metabolism and cell survival in PrCa cells. Effect of Cuc D on protein levels of PI3K, pAKTSer473, c-Myc and phospho-mTOR in DU145 cells as determined by western blot analysis. Briefly, cells were treated with denoted concentrations of Cuc D for 24 h, total cell lysates were prepared and subjected for western blot analysis. α-tubulin was used as an internal loading control. Values shown above the blots are densitometric analysis of PI3K, c-Myc with α-tubulin and ratio of phospho-Akt/total Akt and phospho-mTOR/total mTOR were normalized with α-tubulin as quantitated by Gel-Quant.

In cancer cells, aberrant expression of PI3K/Akt/mTOR signaling pathway has been associated with enhanced metabolic activities including enhanced uptake of nutrients such as glucose, amino acid [21,22,23,24]. It has been reported that AKT activation is associated with glycolysis stimulation in tumor cells [25]. Further, mTOR is thought to have an important role in metabolic regulation which is indirectly activated by AKT [26]. Therefore, we investigated whether Cuc D inhibits the expression of key effector molecules of these signaling pathways in PrCa cells. Our results demonstrated that Cuc D treatment inhibits the expression of PI3K, c-Myc, AKT and mTOR phosphorylation in a dose-dependent manner in DU145 (Figure 5) cells. Collectively, these results suggest that Cuc D may affect glucose metabolism in PrCa via modulating the aforementioned signaling pathways.

### 2.8. Cuc D Inhibits PrCa Cell Derived Xenograft Tumors in Athymic Nude Mice 

**Figure 6:**
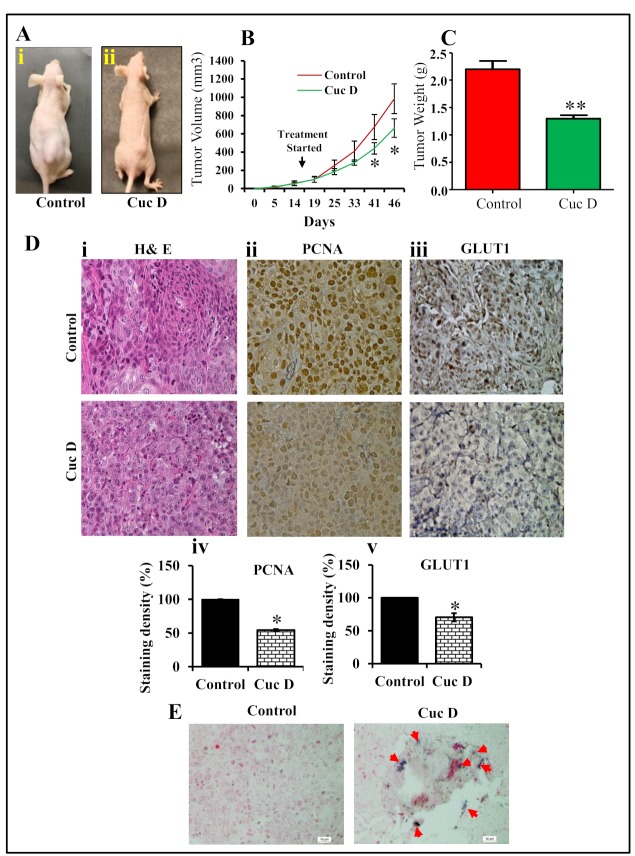
Effect of Cuc D on PrCa cell-derived xenograft tumors in athymic nude mice. (**A**) Representative photograph of athymic nude mice bearing DU145 cells derived xenograft tumors in control (**Ai**) and Cuc D (**Aii**) after 7 weeks. A total of 12 mice were used and divided in two groups: control (*n* = 6) and Cuc D (*n* = 6). DU145 cells (4 × 10^6^) were implanted into right flank of each mouse. Cuc D treatment (1 mg/kg) injected 3 days per week after cell implantation and continued until 7 weeks. Control group mice received 0.2 mL PBS. All mice were sacrificed at 7 weeks when control mice tumor reached at targeted volume of ~1000mm^3^. (**B**) Line graph represents regression of xenograft tumors volume in Cuc D treated mice as compared to control group. Asterisk indicate statistical significance determined by Student’s *t*-test (* *p* < 0.05). (**C**) Bar graph indicates mean of excised tumors weight of control and Cuc D treated mice. Values in bar graph represent mean ± SE 5 mice tumors in each group. Asterisk indicate statistical significance determined by Student’s *t*-test (** *p* < 0.01). (**D**) Illustrative H&E staining images of control and Cuc D treated excised tumors (**Di**). Effect of Cuc D on expression of PCNA (**Dii**) and GLUT1 (**Diii**) in excised tumor tissues of control and Cuc D treated mice as determined by immunohistochemistry using specific antibodies (40×). Densitometry analysis of PCNA (**Div**) and GLUT1 (**Dv**). Asterisk indicate statistical significance determined by Student’s *t*-test (* *p* < 0.05 and ** *p* < 0.01). (**E**) Effect of Cuc D on the expression of miR-132 in control and treated mice excised tumors as determined by in situ hybridization (20×).

To investigate whether Cuc D treatment regress prostate tumor growth, we developed mouse model using DU145 cells. In this study, a total of 12 athymic nude mice were used to develop PrCa cell-derived xenograft tumors. Once the tumor volumes reached 100 mm^3^, mice were divided into two groups as explained in materials and methods. Intra-tumoral injection of Cuc D (1 mg/kg body weight) significantly (*p* < 0.05) regressed xenograft tumors in athymic nude mice as compared to vehicle treated group (Figure 6A). Tumor volume was monitored at regular intervals of time as shown in Figure 6B. Interestingly, Cuc D treated group demonstrated inhibition of tumor growth (Figure 6C). Moreover, we determined the effect of Cuc D on the expression of PCNA and GLUT1. Our results showed a significant decrease in the expression of PCNA in Cuc D treated mice xenograft tumors as compared to control tumors (Figure 6Dii). In addition, there was a decreased in the expression of GLUT1 in Cuc D treated xenograft tumors (Figure 6Diii). The tumor tissues were analyzed for the expression of miR-132 by in situ hybridization (ISH). Interestingly, we found increased expression of miR-132 in Cuc D treated excised xenograft tumors when compared with vehicle groups (Figure 6E). These results further confirm anti-tumor efficacy of Cuc D via inhibiting GLUT1 expression and subsequently restoration of miR-132 in in vivo PrCa model.

## 3. Discussion 

By their nature, cancer cells undergo a higher consumption of biofuels to operate their oncogenic machinery. This rewired metabolism is acquired to support their rapid proliferation and metastasis across the body [27,28]. Drug resistance and systemic toxicity are main limiting factors for successful management of PrCa. Therefore, there is an urgent need for biologically safe and effective natural compounds as anti-cancer drugs. Herein, our study delineates the therapeutic efficacy of Cuc D against PrCa via in reprogramming metabolic shift and molecular interaction with GLUT1 receptor. 

Cucurbitacins have been identified as potent inhibitors of the JAK/STAT pathway [15]. Our findings suggest Cuc D treatment (0–1 µM) induced a marked anti-proliferative effect in PrCa cells (PC3 and DU145). The inhibitory activity of Cuc D on cancer cell growth was also confirmed with a clonogenic assay, indicating that inhibition is irreversible in nature. It also revealed that Cuc D treatment enhanced Annexin V staining and cleavage of PARP protein in PrCa cells, suggesting that inhibitory activity likely occurs via an apoptosis mediated effect. Furthermore, Cuc D arrests the cell cycle progression in G2/M phase. It has been reported that cyclin-dependent kinases (CDKs) activity is regulated by CDK inhibitors such as p21 and p27 families of proteins. We observed, an upregulation of p21 and p27 proteins in PrCa following Cuc D treatment. These findings suggest that Cuc D arrests cell cycle progression in PrCa via modulating cell cycle regulatory proteins.

Furthermore, agents that inhibit the migration and invasion of cancer cells could be used for the prevention and treatment of metastatic cancer. Interestingly, we found that non-toxic doses of Cuc D significantly inhibit the migration of PrCa cells, which represents that Cuc D could be an effective agent to inhibit PrCa cell metastasis. Moreover, our findings demonstrated that Cuc D (0.5 µM) efficiently reduced the invasion of PrCa cells. Our functional experiments show that non-toxic doses of Cuc D significantly decrease migration and invasiveness of PrCa cells. These findings suggest that Cuc D can also be used to inhibit PrCa cell metastasis. 

Accumulating evidence also suggests that glucose scarcity is sufficient to induce growth inhibition and cell death in cancer cells [29,30,31]. A number of natural compounds have shown the ability to modify glucose uptake by regulating GLUT1 expression and/or altering glucose binding to them [12]. Our results demonstrate inhibition of glucose uptake and lactate production by Cuc D in PrCa which provide us a clue that Cuc D may reprogram glucose metabolism to inhibit the growth of metastatic PrCa cells. Notably, western blot analysis showed that Cuc D decreases the expression of GLUT1. Thus, an increase in glucose uptake has been associated mainly with GLUT1 overexpression. Recently, increased expression of GLUT1 has been reported in cancer cells but the mechanism of their aberrant expression is not yet clear [32]. MiR-132 has been shown to modulate the metabolic flux of PrCa by direct targeting GLUT1 [20]. Strikingly, our findings show that Cuc D treatment restores the expression of tumor suppressor miR-132. Furthermore, we observed that miR-132 inhibitor upregulates GLUT1 expression in Cuc D treated PrCa cells, suggesting that Cuc D induces its effect via miR-132 restoration. This is in consistent with other studies where natural compounds have been reported to modulate several epigenetic modification processes known to underlie the molecular mechanism involved in tumorigenesis such as non-coding microRNA expression [33,34,35,36]. To gain further insight, we performed molecular docking analysis by Autodock. Our docking results showed that ligand Cuc D proficiently binds with GLUT1 and free energy for this complex was −8.5 kcal/mol. It showed hydrogen bonding with THR137, SER80 and ARG153 of GLUT1 whereas ALA392, VAL83, IL404, HIS160, GLY408 and TRP388 residues which are responsible for hydrophobic interactions. It may be possible that Cuc D degrades GLUT1 via binding at these residues. However, further studies are warranted to confirm these results. 

The PI3K/AKT/mTOR signaling pathways are highly conserved, which are activated by various growth factors in cells [37]. Activation of these signaling cascades in cancer cells enhance various metabolic activities that are required for cellular biosynthesis [21,22,23,24,38]. AKT enhances glycolysis and lactate production and is adequate to induce the Warburg effect [25,39,40]. Our results also show the inhibition of PI3K, pAKTSer473, and p-mTOR proteins in PrCa cells, which suggests that Cuc D has potential to suppress PI3K/AKT signaling pathways in PrCa cells. In a similar study, He et al. (2017) reported that cucurbitacin E inhibited the mTOR expression in human prostate cancer cells [41]. These results suggest that Cuc D can reprogram glucose metabolism via targeting these signaling components in PrCa cells. 

To translate our in vitro findings into in vivo, we determined the therapeutic efficacy of Cuc D using an athymic nude mice model bearing DU145 cell-derived xenograft tumors. This study showed that intra-tumoral administration of Cuc D (1 mg/kg body weight) inhibited xenograft tumors in athymic nude mice. We did not observe any apparent toxicity in any of the Cuc D administered mouse. These results clearly indicate that Cuc D has potential to inhibit human PrCa cell-derived xenograft tumors. This is in consistent with other cucurbitacin analogue which also exhibited the potent anti-tumor activity in other cancer [42,43]. Further, it showed down-regulation of PCNA and GLUT1 proteins in excised xenograft tumor tissues. ISH results demonstrated that Cuc D treatment restores the expression of miR-132 in excised xenograft tumors. These results indicated that Cuc D replenished the tumor suppressor miR-132 in vitro as well as in vivo.

## 4. Materials and Methods

### 4.1. Cell Culture

The human prostate cancer cells (PC3 and DU145) were a generous donation from Dr. Rajesh Singh (Morehouse School of Medicine, Atlanta, GA, USA). They were procured from ATCC in January 2016. Once received, cells were expanded and stored in liquid nitrogen (passage < 6). For carrying out experiments, cells were thawed and grown for less than 6 months. These cell lines were cultured in RPMI 1640 (HyClone Laboratories, Inc., Logan, UT, USA) and supplemented with 10% heat-inactivated FBS (Atlanta Biologicals, Atlanta, GA, USA), 1% penicillin, and 1% streptomycin (Gibco BRL, Grand Island, NY, USA). 

Cucurbitacin D was obtained from Dr. Fathi T. Halaweish (SDSU, Brookings, SD, USA). Detailed procedure for synthesis and characterization of cucurbitacin D was described previously [44].

### 4.2. Cell Proliferation Assay

The effect of Cuc D on PC3 and DU145 cells proliferation was performed using the MTT assay. Briefly, cells were seeded at a density of 5 × 10^3^ cells per well in 96 well plate and allowed to stand for overnight at 37 °C and 5% CO_2_ incubator. Next day, cells were treated with different concentration of Cuc D (0.1, 0.5 and 1 µM). DMSO was used as vehicle for the treatment of control cells. Fourty eight hours post-treatment, 20 µL of MTT reagent (5 mg/mL) was added in each well and further incubated the plate for 2 h in CO_2_ incubator. Absorbance was taken after 2 h at 570 nm (SpectraMax M2 spectrophotometer, Molecular Devices, Sunnyvale, CA, USA). The experiment was performed in triplicates. Results were represented as percent viability with respect to the control group.

### 4.3. Cell Proliferation by xCELLigence Assay

PrCa cells (10,000 cells per well) were seeded in E-plate following the xCELLigence Real Time Cell Analyzer (RTCA) DP instrument manual as provided by the manufacturer [45]. Average baseline cell index for Cuc D treated cells compared to control cells was calculated.

### 4.4. Colony Formation Assay

To determine the effect of Cuc D on clonogenic potential of PC3 and DU145 cells, colony formation assay was performed. In brief, 500 cells were seeded per well in 6- well plate and allowed to stand for next three days. The cells were treated with Cuc D with different concentrations (25 and 50 nM) for seven days. DMSO was used as vehicle for the treatment of control cells. Colonies were fixed in methanol, stained with haematoxylin, and counted using UVP 810 software.

### 4.5. Apoptosis Analysis

The apoptosis inducing effect of Cuc D on PrCa cells was analyzed by Annexin V-FLUOS staining kit (Roche Diagnostic Corp. Indianapolis, IN, USA). All of the procedure was followed as described in vendor’s protocol. Briefly, 60% confluent PrCa cells (PC3 and DU145) cells were treated with Cuc D (0.5 µM) and kept in CO_2_ incubator at 37 °C for 24 h. Control group cells were treated with DMSO as vehicle. Cells were washed with PBS (1×) and kept in Annexin-V solution for 20 min. Images were captured in bright and green field by fluorescent microscope.

### 4.6. Cell Migration Assay

Cells motility was performed in vitro scratch wound assay. Briefly, cells were seeded in a 12-wells plate and after 80–90% confluency a standardized wound was made using a 200 µL micropipette tip. Cells were then treated with Cuc D (0.25 and 0.5 µM) and photographed at 0 and 48 h by phase contrast microscopy.

### 4.7. Agarose Bead Assay

Cells migration was performed by agarose bead- assay as described earlier [46]. Briefly, cells were mixed into a low melting point agarose solution and drops of suspension were placed onto plates. Cells were treated with Cuc D at 0 and 48 h and the plates were photographed using a phase-contrast microscope.

### 4.8. Cell Invasion Assay

Cell invasion assay was performed using BD Biocoat Matrigel Invasion Chambers (BD Biosciences, San Jose, CA, USA), as described earlier [3]. Cells were treated with Cuc D (0.25 µM) followed by incubation for 18 h. Cells were fixed using methanol and were stained with crystal violet. The images were captured at 18 h.

### 4.9. Cell Cycle Analysis

The effect of Cuc D on cell cycle analysis was performed by flow cytometry as described earlier [17]. In brief, approximately 70% confluent PrCa cells were treated with Cuc D (0.5 and 1 µM) for 24 h. The cells were trypsinized and washed twice with ice-cold PBS (1×). The cell pellets were resuspended in 50 µL ice cold PBS (1×) and 450 µL cold methanol. The cells were washed twice with ice cold PBS (1×), suspended 500 µL PBS and incubated with 500 µL RNase (20 µg/mL final concentration) at 37 °C for 1 hr. The cells were chilled over ice for 10 min and stained with propidium iodide (50 µg/mL final concentration) for 1 h and analyzed by flow cytometry (BD Accuri C6; Becton Dickinson, Mountain View, CA, USA). Data was analyzed by using Modfit software.

### 4.10. Western Blot Analysis

The effect of Cuc D on protein expression in prostate cancer cells were determined by Western blot analysis by using specific antibodies of GLUT1 (cat. no. 12939), c-Myc (cat. no. 9402), pAKTser473 (cat. no. 4060), α-tubulin (cat. no. 2144), p21 (cat. no. 2947), p27 (cat. no. 3686), PI3K110 (cat. no. 4249), PARP (cat. no. 9542), PCNA (cat. no. 2586), Phospho-mTOR (Ser2448) (cat. no. 2971) were obtained from Cell Signaling Technology Inc. (Danvers, MA, USA) Horseradish peroxidase (HRP)-conjugated anti-mouse (cat. no. 4021) and anti-rabbit (cat. no. 4011) antibodies were acquired from Promega (Madison, WI, USA). 

### 4.11. Isolation of RNA and PCR

RNA from prostate cancer cells was isolated using Qiagen kit and quantified using NanoDrop instrument 2000 (Thermo Scientific, Waltham, MA, USA). To analyze the expression of miR-132 in control and Cuc D treated cells, 100 ng total RNA was reverse transcribed into cDNA using specific primers designed for miRNA analysis (Applied Biosystems, Foster City, CA, USA). The expression of this miRNAs was determined by qRT-PCR using the Taqman PCR master mixture (no AmpErase UNG) and specific primers designed for detection of mature miRNAs (Applied Biosystems). The expression of miRNA was normalized with the expression of endogenous control, RNU6B.

### 4.12. Glucose and Lactate Assay

Glucose and lactate assays were performed using kits (#10009582 and #600450, Cayman Chemicals, Ann Arbor, MI, USA. Prostate cancer cells were seeded (10^4^ cells/well in 96-well plate) and media collected to measure the amount of lactate after 24 h, and unused glucose levels after 48 h. The samples were analyzed according to the instructions provided in the kit, the readings were recorded, and calculations were done. The fluorescent D-glucose analog 2-(N-(7-nitrobenz-2-oxa-1,3-diazol-4-yl)amino)-2-deoxyglucose (2-NBDG) was used as a fluorescent indicator to evaluate glucose update in PrCa cells. Cells were treated with Cuc D (0.25 and 0.5 µM) followed by 2-NBDG and images were captured by fluorescence microscope.

### 4.13. Xenograft Study

Next, we assessed the anticancer activity of Cuc D in ectopic xenograft mouse model of PrCa. Twelve 6-week old male athymic nude mice (Jackson laboratory, Bar Harbor, ME, USA) were used. Mice were maintained in a pathogen-free environment and all the procedures were carried out as per the protocol approved by the UTHSC Institutional Animal Care and Use Committee (UTHSC-IACUC; protocol number 17-038). Briefly, a mixture of equal volume of DU145 cells (4 × 10^6^) and 100 µL matrigel (BD Biosciences) was injected subcutaneously on the dorsal surface of each mouse. Once the tumor volume reached ~100 mm^3^, Cuc D (1 mg/kg) and the respective vehicle control (1× PBS) were administered by intra-tumoral injection three times a week for 5 weeks. The tumor volume was periodically measured using the ellipsoid volume formula: tumor volume (mm^3^) = 0.5 × *L* × *W* × *H*, wherein *L* is length, *W* is width, and *H* is height. Once the endpoint is reached, mice were sacrificed, and their tumors were excised and used for tissue sectioning (5-micron) for histopathology and biochemical analyses.

### 4.14. Immunohistochemistry (IHC)

The effect of Cuc D was determined on proliferating cell nuclear antigen (PCNA) and GLUT1 proteins in excised tumors by immnunohistochemistry using Biocare kits (Biocare Medical, Concord, CA, USA). Briefly, the tumor tissues were deparaffinized, rehydrated followed by antigen retrieval using a heat induced technique. Samples were incubated overnight for staining with PCNA and GLUT1 antibodies. The slides were counterstained with hematoxylin, followed by mounting with vecta mount (Vector Laboratories, Burlingame, CA, USA) and visualized.

### 4.15. In Situ Hybridization for miR-132

The expression of miR-132 in FFPE tissues of control and treated xenograft mice was determined by in situ hybridization analysis using Biochain kit (catalog number K2191050; Biochain IsHyb In Situ hybridization kit) according to the manufacturer’s protocol. Briefly, tissues were deparaffinized and fixed in 4% paraformaldehyde and DEPC-PBS for 20 min. They were subjected to digestion using 2× SSC and 0.1% triton X for next 25 min. The tissues were prehybridized with prehybridized solution provided with the kit for 4 h at 48 °C. This followed the hybridization of the slides with hybridization buffer and digoxigenin labelled probe (Exicon, Woburn, MA, USA) at 45 °C overnight. After stringent washing of tissue slide with various grades of SSC, the slides were blocked using 1× blocking solution provided with the kit. This followed the subsequent incubation of tissues overnight with AP-conjugated anti-digoxigenin antibody. Further, the slides were washed for 5 min with 1× alkaline phosphatase buffer twice. The final visualization was carried out with NBT/BCIP (Pierce, Rodkford, IL, USA) followed by nuclear fast red counterstaining. The slides were mounted and analyzed under microscope.

### 4.16. Molecular Docking

The 2D and 3D structures of Cuc D were retrieved from PubChem. Atomic coordinates for the crystal structure of GLUT1 (PDB ID: 4PYP) were taken from Protein Data Bank (www.rcsb.org). Other calculations and file preparations were done according to our published protocol [47]. By using standard protocol of AutoDock 4 package, Cuc D was docked into binding site of GLUT1 [48]. To deal interactions which exists between GLUT1 and Cuc D, the Lamarckian genetic algorithm (LGA) was applied [49,50]. Polar hydrogen atoms were added geometrically. Final docked complexes were optimized, validated and analyzed using “Receptor–Ligand Interactions” modules present in the script section of Discover Studio 4.0. Further to visualize molecular interactions, resultant dock structure files were analyzed by PyMOL [51].

### 4.17. Statistical Analysis

Statistical analysis was performed using an unpaired two-tailed Student *t*-test and employed to assess the statistical significance between the control and Cuc D treated groups. 

## 5. Conclusions

In summary, this study revealed a novel therapeutic role of Cuc D in modulating glucose metabolism and inhibits oncogenic signaling pathways in PrCa. Our study offers very promising findings that could be a novel chemotherapeutic agent alone or in combination with ongoing chemotherapy regimen for the management of advanced PrCa.
